# Unpack the Salt: an evaluation of the Victorian Salt Reduction Partnership’s media advocacy activities to highlight the salt content of different foods

**DOI:** 10.1186/s12937-020-00621-0

**Published:** 2020-09-16

**Authors:** Emalie Rosewarne, Kathy Trieu, Clare Farrand, Jenny Reimers, Jane Potter, Chelsea Davidson, Natasha Darrigan, Elizabeth Joldeski, Sian Armstrong, Jacqui Webster

**Affiliations:** 1 The George Institute for Global Health, The University of New South Wales, Sydney, NSW 2006 Australia; 2 Victorian Health Promotion Foundation, Melbourne, VIC 3053 Australia; 3 National Heart Foundation of Australia, Melbourne, VIC 3008 Australia

**Keywords:** Media advocacy, Salt reduction, Australia

## Abstract

**Background:**

Australians are consuming almost double the recommended maximum salt intake. The Victorian Salt Reduction Partnership was established to coordinate efforts to reduce salt intake in the state of Victoria. As part of an intervention strategy, media advocacy strategies were used to raise public awareness and stimulate industry and government action on salt reduction. This study aimed to evaluate the Victorian Salt Reduction Partnership’s media advocacy activities by determining the extent to which activities contributed to the overall strategy aims and the effectiveness of the activities in gaining media and industry engagement.

**Methods:**

A framework for evaluating media advocacy strategies used in complex public health interventions was used to guide this evaluation. Media advocacy activities were monitored and documented throughout the intervention period. A content analysis of media release press statements was performed. Indicators of media coverage (media items, cumulative audience reach, advertising space rate) and food industry engagement (number of meetings, number and type of follow up actions) were tracked.

**Results:**

Six media releases were issued between March 2017 and November 2018 on different processed food categories including breads, cooking sauces, ready meals, dips and crackers, processed meats and Asian-style sauces. Three main themes were identified in the qualitative analysis of the press statements: general information on salt and health, salt levels in foods, and calls to action for consumers, industry and/or government. These themes were aligned with the overall intervention strategy. Media items (print and online news, radio and TV) generated by each release ranged from 36 to 274, and cumulative audience reach (opportunities to see) ranged from 2.3 to 7.5 million Australians per release. One to three food manufacturers were met with per media release.

**Conclusions:**

Disseminating sodium-monitoring data through media releases can be used as a tool to gain access to the media and reach consumers with salt reduction messages, and to engage food manufacturers in discussions about salt reduction. Characteristics of media advocacy activities, including alignment with the overall strategy, and external factors outside the of control of the program implementers, can influence media and industry engagement. When planning future nutrition interventions that include media advocacy activities, internal and external factors impacting outcomes, should be considered, documented and evaluated.

## Background

Excess dietary sodium intake increases blood pressure, and consequently increases the risk of cardiovascular diseases [1]. Dietary sodium is usually consumed as sodium chloride, or salt [2], and in 2017, 3.2 million global deaths were attributable to high salt diets [3]. To reduce the disease burden from excess salt consumption, the World Health Organization (WHO) recommends reducing population salt intake to less than 5 g per day [4].

For Australians, mean salt intake is estimated at 9.6 g/day [5], which is almost double the WHO recommendation [4]. Despite the global target to reduce salt intake by 30% by 2025, concerted action to reduce salt intake in Australia is lacking [2, 6]. In response, the Victorian Salt Reduction Partnership (VSRP) was established in 2014 to coordinate efforts to reduce salt intake in the state of Victoria. The VSRP comprises key organisations working on salt reduction including the Victorian Health Promotion Foundation (VicHealth), The George Institute for Global Health, The Heart Foundation, Deakin University and the Victorian Department of Health and Human Services [7]. The overall aim was to reduce the average salt intake of Victorians by 1 g per day by 2020 [7].

In Victoria, it is estimated that more than 80% of dietary salt is from processed and ultra-processed foods [8]. As such, the key focus of the VSRP strategy has been on reducing salt intake from processed foods. In 2017, the VSRP launched the *Unpack the Salt* campaign. The three main intervention components were: raising public awareness of the need to reduce salt intake and of salt levels in foods; stimulating industry action to reformulate foods to contain less salt; and advocating for the federal government to set targets for sodium levels in foods [7, 9]. To support the delivery of each of these components, product category reports utilising sodium-monitoring data from the FoodSwitch database were produced by The George Institute for Global Health [10, 11]. These reports assessed the mean salt content of different product categories, and variations in salt levels within categories. Product categories were selected by the VSRP if they were one of the highest contributors of salt to the diet or were a high salt category with a high frequency of consumption, as determined by the most recent Australian Health Survey [12], as reformulation of these product categories offers an important opportunity to reduce population level salt intake. The product category reports have been previously published and the main results are presented in Supplementary Table 1. The key findings were translated into a press statement and communicated via periodically scheduled media releases throughout the intervention timeframe [10]. The dissemination strategy utilised both public awareness raising and media advocacy approaches. Public awareness components created opportunities for consumers to improve their health literacy and aimed to empower consumers to take action. Media advocacy (i.e. the innovative and strategic utilisation of mass media as a tool for health promotion [13]) was used to stimulate industry action to reformulate and government action to set sodium targets, as well as increase public support for these actions. Given most Australian food manufacturers are national and international companies, and that food policies are enacted by the federal government, media advocacy activities were executed, and expected to have an impact, at both the state and national level.

The objectives of this research were to document implementation of media advocacy activities and evaluate the VSRP media advocacy strategy. The evaluation included determining the extent to which activities contributed to the overall VSRP strategy aims and the effectiveness of the activities in gaining media and industry engagement. This study is a component of an ongoing comprehensive process evaluation of the VSRP strategy [9].

## Methods

Stead et al.’s framework for evaluating media advocacy strategies used in complex public health interventions was used to guide the qualitative and quantitative analysis [14]. The research methods included: (1) monitoring and audit of media advocacy activities and descriptive analysis of press statements to document implementation; (2) content analysis to understand the extent to which the media advocacy activities contributed to the overall VSRP strategy aims; and (3) descriptive quantitative analysis of media coverage and industry engagement to assess the effectiveness of media advocacy activities in gaining access to the media and food industry.

### Monitoring and audit of media advocacy activities

Media advocacy activities were documented throughout the intervention period by the Heart Foundation. Details recorded included the number of media releases and additional material provided in the media releases (e.g. infographics). The headline and lead message were extracted from each press statement to illustrate the framing of the media release.

### Qualitative analysis of the media advocacy strategy

A content analysis of the press statements was performed to identify the key characteristics of the media advocacy activities. Using *NVivo* for data management, themes of each press statement were identified inductively through line-by-line analysis [15]. Identified themes were mapped to VSRP process evaluation logic model [9] to understand the extent to which the activities contributed to the overall VSRP strategy [14].

### Descriptive quantitative analysis of the media advocacy strategy

#### Indicators of media coverage

Indicators of national media coverage were tracked by the Heart Foundation. Indicators included: the number of media items generated by the press statements, overall and by type of media (e.g. radio, online news); cumulative audience reach (opportunities to see; the potential number of unique individuals reached through an advertising medium over a specified time); advertising space rate (ASR; the estimated dollar equivalent of buying advertising space in media, which indicates the prominence of media items); and use of the *Unpack the Salt* hashtag.

#### Indicators of industry engagement

Food manufacturers named in each media release as having the highest or lowest salt products were contacted by the Heart Foundation, via email communication or phone call, 24-h prior to the media release to: notify them of the results of the report, give them an opportunity to prepare comments in response and to propose future discussions on reformulation of high salt products.

To quantify industry engagement the following information was recorded by the Heart Foundation for each media release: number of food manufacturers who were contacted by, or contacted, the Heart Foundation or VSRP; the number of initial and follow up meetings; or any follow up actions. Follow up actions included: referral to other resources, engagement in product benchmarking services by The George Institute for Global Health, or completion of a commitment statement or company case study on salt reduction, which could then be published on the *Unpack the Salt* website.

## Results

### Monitoring and audit of media advocacy activities

Six media releases were issued between March 2017 and November 2018. The media releases comprised: the product category report [16–21], a press statement [22–27], key messages as infographics [28–32], and a list of products that had the lowest and highest amounts of salt in each category [33–38]. The product categories included were breads, cooking sauces, ready meals, dips and crackers, processed meat and Asian-style sauces (Table 1).

**Table 1:** Overview of each media release obtained from the press statement

Product category	Release date	Headline of press statement	Lead message from press statement
Bread [22]	21-Mar-17	Victorians urged to curb their consumption of salt	“some loaves contained more than a third of the daily recommended salt intake in just two slices.”
Cooking Sauces [23]	23-Aug-17	New research shows family favourite cooking sauces are packed with salt	“they’re packed with salt, with some brands nearly 100 times worse than others”
Ready Meals [24]	3-Oct-17	Australian ready meals are saltier than ever	“some ready meals contain more than an entire day’s worth of salt in a single serve and … they’re getting saltier”
Dips and Crackers [25]	7-Dec-17	Research reveals that many healthy-looking dips are saltier than seawater	“some dips are saltier than seawater, and several cracker-dip combinations deliver more than half a day’s worth of salt in just one serve.”
Processed meats [26]	14-Mar-18	Aussie BBQ classic proving a snag to good health	“the humble snag in white bread and tomato sauce contains a whopping 2.35 g of salt – nearly half of the recommended daily salt intake”
Asian-style sauces [27]	13-Nov-18	Hold the sauce: New report finds swapping your soy sauce can halve your salt intake	“a single tablespoon of the average soy sauce contains 61% of our recommended daily salt intake”

The headline and lead message from the press statement illustrate the framing of each report [22–27]. The headline for three of six reports stated the high salt content of the food category (cooking sauces, ready meals, dips and crackers), while two advised consumers to reduce salt intake (bread, Asian-style sauces) and the other didn’t mention salt but rather good health (processed meats). The lead message for five of six reports compared consumption of a food product to the recommended daily maximum salt intake, while the cooking sauces lead message compared salt levels within the product category (Table 1).

### Qualitative analysis of the advocacy strategy

Three main components of the press statements were identified in the content analysis: general salt information such as current population salt intakes and the link between high salt intake and disease/health outcomes (4 themes); key findings from the product category report including salt levels of certain products and categories (4 themes); and a call to action for consumers, industry and/or government (3 themes; Table 2). All of these contribute to the main aims of the media strategy: (1) to raise public awareness of the need to reduce salt intake and salt levels in foods, (2) to stimulate industry action to reformulate foods to contain less salt and (3) to persuade the federal government to set targets for sodium levels in foods.

**Table 2:**
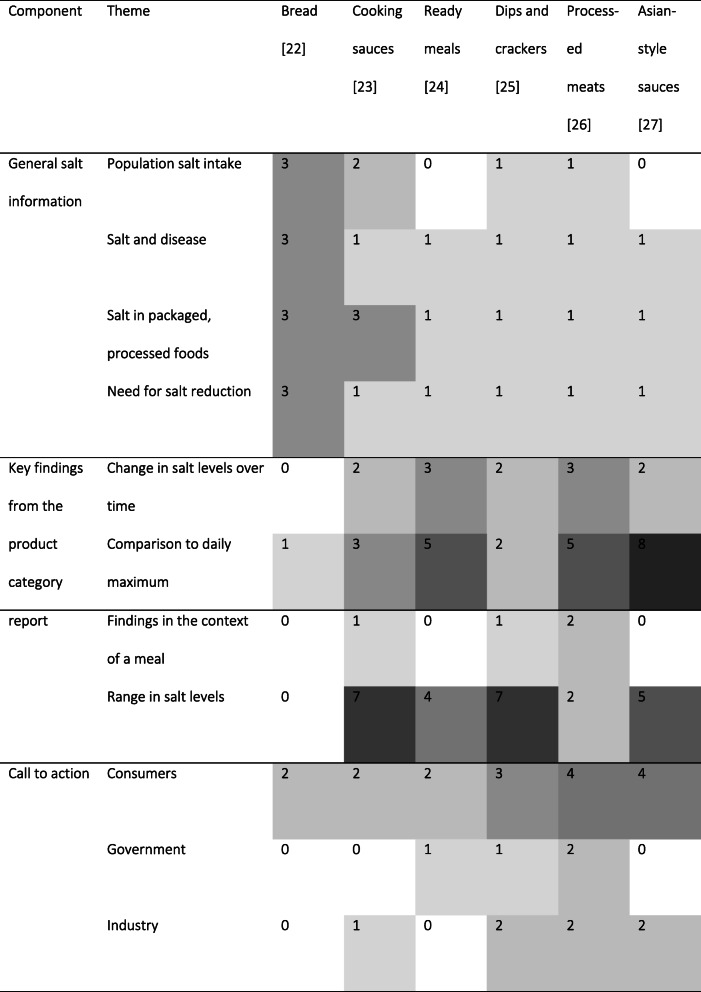
Main themes identified in the press statements and frequency of occurrence by product category

All press statements included general salt information in line with the VSRP strategy, including information on the burden of disease attributable to high salt intakes and the need for salt reduction in Australia, although only four of six mentioned current salt intake levels in Australia. Every press statement outlined that the majority of salt in the Australian diet is from packaged and processed foods (Table 2).

All press statements included key findings from the product category report, including comparing salt levels in products to the recommended daily maximum amount of 5 g per day. All, except bread, highlighted the range in salt content between sub-categories, and/or highest and lowest salt products overall. Similarly, all except bread, mentioned changes in salt levels over time. Three of six press statements, cooking sauces, dips and crackers, and processed meats, contextualised the food product as part of a meal (Table 2).

Every press statement called on consumers to take action to reduce their salt intake, while four of six called on industry to take action to reformulate foods to include less salt and three of six called on government to set sodium targets for manufacturers to meet. Only two, dips and crackers and processed meats, included all three call to action messages (Table 2).

### Indicators of media coverage and industry engagement

A total of 759 media items were identified across the media releases measured (excluding bread). The number of media items ranged from 36 items for dips and crackers to 274 items for processed meats. Media coverage ranged from an estimated cumulative audience reach of 2.3 million (dips and crackers) to 7.5 million (processed meats). The highest ASR was achieved for processed meats at $1,633,188, followed by Asian-style soy sauces at $1,105,824. Social media engagement through use of the *Unpack the Salt* hashtag ranged from 63 (dips and crackers) to 143 (processed meats; Table 3).

**Table 3:** Indicators of media coverage and industry engagement in relation to the six media releases

	Bread^**a**^	Cooking Sauces	Ready Meals	Dips and Crackers^**c**^	Processed meats	Asian-style sauces
**Indicators of media coverage**						
**Total media items**	N/A	181	137	36	274	131
AM Radio	N/A	17	30	6	19	23
FM Radio	N/A	10	12	0	15	11
Newspaper	N/A	3	7	6	11	1
Online news	N/A	123	67	21	185	65
TV	N/A	28	21	3	44	31
**Total cumulative audience reach**	956,031^b^	6,582,596	5,884,395	2,316,478	7,460,925	5,987,529
AM Radio	N/A	1,931,100	1,523,200	900,000	680,500	1,867,200
FM Radio	N/A	56,800	175,000	0	338,500	168,100
Newspaper	N/A	421,452	281,393	337,745	829,136	5535
Online news	N/A	10,244	51,802	50,733	114,689	98,757
Television	N/A	4,163,000	3,853,000	1,028,000	5,498,100	3,848,000
**Total advertising space rate**	N/A	$961,188	$1,013,613	N/A	$1,633,118	$1,105,824
AM Radio	N/A	$225,508	$77,715	N/A	$49,286	$63,226
FM Radio	N/A	$1752	$34,138	N/A	$14,819	$30,308
Newspaper	N/A	$254,952	$72,217	N/A	$124,383	$1405
Online news	N/A	$75,150	$169,881	N/A	$576,498	$494,457
TV	N/A	$403,826	$682,849	N/A	$868,132	$516,428
**Social media engagement #UnpackTheSalt**	N/A	96	80	63	143	112
**Indicators of industry engagement**						
**Number of manufacturers contacted by VSRP**	N/A	13	5	11	2	8
**Number of manufacturers who contacted VSRP**	1	0	0	0	0	0
**Number of manufacturers meeting with VSRP/direct engagement**	1	3	2	1	2	2
**Number of manufacturers with follow up meetings with VSRP**	0	2	2	1	2	0
**Number of manufacturers engaged in follow up activities**	0	2	1	1	2	2
Referral to other resources, networks or people e.g. state government, international work	0	2	1	1	2	2
Number of manufacturers that completed a VSRP commitment statement	0	0	0	0	0	0
Number of manufacturers producing a case study	0	2	1	0	0	0
Number of manufacturers engaged in benchmarking services	0	1	1	0	0	0

*VSRP* Victorian Salt Reduction Partnership

^a^ The bread product category report was released prior to the launch of the Unpack the Salt campaign. Media indicator data for the bread report were collected by The George Institute for Global Health, whereas data for other product category reports were collected by the Heart Foundation

^b^ Average audience reach, rather than cumulative audience reach, was recorded for the bread product category report

^c^ Advertising space rate data was not collected for dips and crackers

For industry engagement, one manufacturer contacted, and met with, the VSRP after the bread media release. For subsequent releases, the number of manufacturers contacted by the VSRP ranged from two to 13. At least one manufacturer was engaged in a meeting, for every product category, though engagement rates varied from 9% for dips and crackers (1/11 manufacturers) to 100% for processed meats (2/2 manufacturers). A total of 10 manufacturers were engaged, including all four major retailers, and three manufacturers in Australia’s top 100 food and drink companies [39]. One manufacturer was met with in relation to two separate product categories. Follow up meetings were held with seven manufacturers and these ranged from one meeting to three meetings (Table 3).

Through industry engagements, seven manufacturers were referred to other resources, networks or people to support them with product reformulation, two manufacturers were engaged in product benchmarking services and three manufacturers produced a case study on their salt reduction reformulation work (Table 3).

## Discussion

Disseminating sodium-monitoring data of the Australian food supply through periodic media releases enabled the VSRP to raise public awareness of the salt content of different foods by gaining access to mass media and engage food manufacturers in meetings to discuss product reformulation. Media releases are a cost-effective way to stimulate reportage of public health issues and reach millions of people with health promotion messaging [40]. Through the six media releases disseminated by the VSRP over a two-year period, more than 750 media items about salt reduction were generated through radio, newspaper, online news, and television. This was much greater than the 58 media items that were generated by six media releases about tobacco control in New South Wales [40], which aimed to increase news coverage of tobacco and health in the media. However, in both cases the number of media items generated by each release varied, from 36 to 274 items for the salt reduction and from zero to 39 items for the tobacco control strategy [40]. Further, the VSRP media coverage was estimated to reach between 2.3 million to 7.5 million Australians per release, approximately 9 to 30% of the population [41]. This variability in media items and coverage between product categories could be the result of characteristics of certain media releases, including alignment with the VSRP strategy [14], or external factors affecting media interest outside of the control of the VSRP [40].

Our qualitative analysis provides insight into the characteristics of the media releases that may have influenced media coverage. Media releases that clearly and concisely explain the public health issue and why it is important have the potential to increase public awareness and influence public opinion and policy change [40]. Every VSRP media release explained the link between salt and disease and the need for Australian’s to reduce salt intake, in line with the VSRP strategy [9]. Each release also highlighted that most salt in the diet was from processed foods and focused on salt levels in one product category. By focusing on one product category, each media release was unique, which likely facilitated continued media interest in salt-related stories over time [40]. These key characteristics likely contributed to the success of the VSRP media advocacy activities in gaining media coverage.

The framing of the media release can also be important for attracting media. Themes and frames that satisfy news values influence media coverage through journalist’s selection of stories that they perceive to be newsworthy [42, 43]. The processed meats release had the highest number of media items, cumulative audience reach, ASR, and social media engagement. This release may have attracted greater media coverage than others due to the framing of the press statement [26], such as the media angle chosen, where the headline mentioned “good health” rather than reducing salt intakes or the high salt content of the food category, or other key findings of interest, for example the reduction in the salt content of bacon but not sausages, over time. However, it could also reflect external factors, such as: the timing of the media release (Salt Awareness Week 2018 [44, 45]), a planned media event in Melbourne with a local chef [46], perceived newsworthiness [40], or general public or media interest in the category.

The dips and crackers release performed the poorest across all media coverage indicators. The press statement [25] was aligned with the VSRP strategy and similar to the other media releases, however the framing was different in that the salt content of dips was compared to seawater. Comparisons, or analogies, are a common framing strategy to gain media and consumer attention for public health issues [47]. Comparing salt levels in specific food categories to the salt content of seawater or crisps have been done by other research and advocacy groups (e.g. [48, 49]) and seems to be an effective strategy for engaging the media. Therefore, the potential reasons for lower media uptake are likely external. The timing of the dips and crackers media release, specifically other events occurring in the world at the time, with the Aztec High School shooting in America occurring on the same day [50], was likely a major factor. Other timing factors, including the lead up to Christmas, other health or nutrition related stories in the week, and the short space of time since the previous media release could have also contributed to the lower media pick-up [40]. The perception from the media that because dips and crackers are discretionary or occasional foods [51] the salt content is a lesser a priority, may also be a factor.

Due to data limitations, we were unable to compare the media indicators for bread with other product categories, however possible factors influencing media outcomes include the focus on general salt information in the press statement rather than the key findings of the product category report (which may have contributed to less media interest [40]), less alignment with the VSRP strategy (e.g. no call to action for industry and government), and the release date, which was before the *Unpack the Salt* campaign launch [23]. It is not always possible predict the contextual factors that will impact media coverage, however for future strategies it is important to consider and anticipate potential influencing factors and document and evaluate impact on media coverage.

Ten companies, including Australia’s four major retailers, three large manufacturers and three small manufacturers were engaged through the six media releases. Although this is a small number of companies, and only a quarter of those contacted by the VSRP, this engagement suggests that media publicity can be used by public health advocates to engage the food industry in discussions about food reformulation. In the UK, this ability to engage the food industry in salt reduction discussions influenced food industry action [45]. Following the cooking sauces media release, as a direct result of engagement with the VSRP, one multi-national food company reformulated a range of cooking sauce products. It is not yet known if other companies reformulated as a result of the VSRP media advocacy activities.

Levels of engagement with food manufacturers differed between reports, which could be due to the VSRP approach to engagement, manufacturer-specific factors, product category-specific factors or the policy environment. The number of manufacturers contacted by the VSRP per report varied depending on how many were named in the release as having the highest salt products [33–38], however, the number of manufacturers engaged per report did not reflect this. Both manufacturers contacted in relation to the processed meat report met with the VSRP compared to only one of 11 manufacturers of dips/crackers. Whether the press releases included a call for industry to act did not seem to influence the likelihood of manufacturers to engage with the VSRP. It is likely that manufacturer-specific factors, such as company philosophy, size and location (overseas, Australia-based), capacity and available resources for reformulation, and receptivity to the industry engagement approach (naming manufacturers and products in the media) influenced manufacturers’ decisions to engage with the VSRP. The feasibility of reformulation for specific product categories is also a likely factor influencing manufacturer engagement. Some foods are harder to reformulate than others due to technical and functional roles of salt, such as its use as a preservative in many products, role in the control of yeast growth and fermentation in bread, and role in sensory and textural properties in processed meats [52]; and manufacturers of these product types may be more likely to be looking for support. Lastly, the lack of sodium targets for these processed foods at the time of these media releases meant that there was no incentive for manufacturers to reformulate other than corporate social responsibility [53]. After the VSRP campaign, in May 2020, the federal government announced the first wave of voluntary sodium targets for manufacturers to meet by 2024, which include four VSRP targeted categories: bread, cooking sauces, crackers (not dips), and selected processed meats [54]. Our results indicate that media advocacy can be used as a tool to engage manufacturers in conversations about salt reduction reformulation, however industry’s responsiveness may depend on a number of factors and in the absence of sodium targets, uptake of VSRP support for reformulation was low.

The idea of utilising product category reports to advocate for salt reduction through mass media stemmed from a similar strategy in the UK, whereby regular surveys were undertaken by Action on Salt, and used to raise public awareness and put pressure on the food industry to reformulate in line with the UK’s salt targets [45]. Between 2006 and 2011, the UK salt reduction strategy reduced population salt intake by 15% and salt levels in foods were reduced by up to 57% in some food categories [45]. Building on concepts from the UK strategy, the VSRP utilised mass media to call consumers, industry and government to act to reduce population salt consumption.

Consumer messages in the press statements were based on evidence about salt levels in different food categories from the product category reports [16–21, 55] and were centred around concepts such as: raising awareness of the salt content of different foods, swapping to a reduced salt variety, replacing processed foods with fresh foods, and trying homemade options. Although the key messages are based on a successful strategy and media coverage indicators seem promising, it is currently unknown whether these messages were enough to trigger changes in consumer behaviour to reduce the salt intake [56]. Media advocacy strategies were used to stimulate industry action to reformulate processed foods and government action to set sodium targets, as well as increase public demand for these actions, in line with the aim to reduce salt in the food supply. In four of six press statements, industry was called to act, with messages highlighting that reformulation is feasible within these product categories, and in three of six press statements, the federal government was also called to act, specifically to set targets for sodium levels in foods.

Worldwide, 61 countries have reported working with the food industry to reformulate products to include less salt [57]. At least 23 countries have reported engaging in industry meetings as part of their national salt reduction strategy [58], the approach undertaken by the VSRP. However only two of these countries, France and Italy, have reported a reduction in the salt levels of selected food categories [58]. Unlike the other 21 countries engaged in industry meetings, these countries also had voluntary sodium targets for bread, which likely influenced their success [58]. In total, 19 of 36 countries that have established sodium targets reported a reduction in salt levels in foods and meals [57]. This emphasises the importance of nutrient reformulation targets for reducing salt levels in the food supply, and consequently decreasing population salt intake and the burden of disease associated with excess salt consumption.

This study is a novel assessment of the outcomes of a media advocacy strategy in Australia. It provides insight into media coverage and industry engagement outcomes from six media releases based on salt levels in different processed food categories. It provides an in-depth understanding of the factors influencing the effectiveness of using media advocacy as a tool for engaging media and industry, which is a key element of a larger salt reduction intervention strategy. The methodology for assessing the media and advocacy strategy, both quantitatively and qualitatively, was based on items from a public health framework for evaluating complex public health interventions [14]. A limitation of the study is that the indicators of media coverage for the bread release were unable to be compared to other media releases. For this report, average audience reach was recorded by The George Institute for Global Health, whereas cumulative audience reach, media items and ASR were collected by the Heart Foundation for the other reports.

## Conclusions

This evaluation of the VSRP media advocacy activities shows that media releases highlighting sodium levels in the Australian food supply can be used as a tool to reach consumers with salt reduction messages through mass media and to engage food manufacturers in discussions about salt reduction strategies. Media and industry outcomes can be influenced by internal and external factors, both of which should be considered when planning future nutrition interventions. However, further research is needed to determine exactly what characteristics or factors increase the effectiveness of media advocacy for nutrition interventions. Continued advocacy efforts calling the federal government to implement and monitor sodium targets and industry to reduce salt in the food supply are needed to reduce population salt intake and raising public awareness can support these interventions and create demand for change.

## Supplementary information

**Additional file 1: Supplementary Table 1.** Mean and range in sodium content of processed foods between 2010 and 2017/18 from six product category reports.

## Data Availability

The datasets used and/or analysed during the current study are available from the corresponding author on reasonable request.

## Abbreviations

WHO: World Health Organization
VSRP: Victorian Salt Reduction Partnership
ASR: Advertising space rate
