# Supplementary material for: Unpack the Salt: an evaluation of the Victorian Salt Reduction Partnership’s media advocacy activities to highlight the salt content of different foods
Source: Nutr J. 2020 Sep 16;19:102. doi: 10.1186/s12937-020-00621-0 (PMC7495894; doi:10.1186/s12937-020-00621-0)
Supplement: Supplementary file 1 — Additional file 1: Supplementary Table 1. Mean and range in sodium content of processed foods between 2010 and 2017/18 from six product category reports. [file 12937_2020_621_MOESM1_ESM.docx]

**Supplementary Table 1. Mean and range in sodium content of processed foods between 2010 and 2017/18 from six product category reports**

|  | 2010 | | | 2013 | | | 2015 | | | 2017/2018^a^ | | | P value | |
| --- | --- | --- | --- | --- | --- | --- | --- | --- | --- | --- | --- | --- | --- | --- |
| Product category | n | Mean (SD, mg/100g) | Range (mg/100g) | n | Mean (SD, mg/100g) | Range (mg/100g) | n | Mean (SD, mg/100g) | Range (mg/100g) | n | Mean (SD, mg/100g) | Range (mg/100g) | ANOVA^b^ | 2010 vs  2017/18^c^ |
| Breads | | | | | | | | | | | | | | |
| Bread and bread rolls | 142 | 456.2 (101.5) | 170 - 770 | 268 | 416.1 (93.1) | 180 - 800 | 249 | 410.1 (87.4) | 40 - 660 | 229 | 407.8 (87.6) | 200 - 880 | <0.001 | <0.001 |
| Flat bread | 46 | 447.7 (218.0) | 110 - 930 | 105 | 562.8 (230.0) | 110 - 1100 | 90 | 542.7 (229.1) | 29 - 1020 | 85 | 363.4 (243.8) | 29 - 930 | 0.23 | 0.55 |
| Morning goods | 23 | 534.4 (135.1) | 360 - 700 | 30 | 492.9 (144.7) | 260 - 674 | 18 | 513.7 (124.9) | 338 - 700 | 21 | 461.3 (151.7) | 229 - 674 | 0.36 | 0.40 |
| Value added bread products | 26 | 522.1 (126.2) | 150 - 668 | 42 | 528.0 (135.1) | 150 - 820 | 40 | 498.6 (106.0) | 270 - 1020 | 28 | 365.5 (119.6) | 174 - 720 | <0.001 | <0.001 |
| Cooking sauces | | | | | | | | | | | | | | |
| Ambient meal-based sauces | 110 | 541.5 (279.7) | 169 - 1600 | 137 | 815.8 (1016.9) | 188 - 5890 | 125 | 780.2 (1072.5) | 108 - 8210 | 100 | 553.8 (412.6) | 120 - 2250 | 0.01 | 1.00 |
| Liquid meal-based sauces | 73 | 2157.4 (881.6) | 132 - 4520 | 83 | 3048.2 (1048.7) | 526 - 11000 | 86 | 1261.9 (947.4) | 118 - 5750 | 58 | 1999.6 (892.8) | 251 - 4530 | 0.45 | 0.62 |
| Powdered meal-based sauces | 112 | 1405.6 (1954.9) | 883 - 12140 | 116 | 1377.1 (2246.0) | 118 - 7050 | 54 | 4543.4 (1972.2) | 507 - 8700 | 112 | 1215.2 (2032.0) | 57 - 4940 | 0.16 | 0.86 |
| Curry pastes | 26 | 5288.2 (1086.9) | 563 - 4660 | 79.0 | 4863 (1947.0) | 298 - 11000 | 59 | 2202.3 (1093.1) | 283 - 5960 | 64 | 5011.3 (897.9) | 715 - 8950 | <0.001 | 0.98 |
| Ambient pasta sauces | 120 | 471.6 (163.5) | 19 - 1200 | 160 | 409.5 (176.0) | 15 - 1200 | 178 | 372.0 (138.4) | 15 - 898 | 145 | 342.0 (146.2) | 8 - 750 | <0.001 | <0.001 |
| Fresh pasta sauces | 15 | 315.0 (116.6) | 0 - 480 | 36 | 323.0 (118.2) | 116 - 660 | 22 | 322.8 (121.6) | 116 - 547 | 21 | 375.9 (101.0) | 190 - 547 | 0.30 | 0.49 |
| Pesto | 9 | 1026.0 (402.1) | 248 - 1540 | 23 | 1011.8 (318.0) | 458 - 1597 | 21 | 1004.9 (338.4) | 466 - 1597 | 20 | 968.6 (341.1) | 259 - 1350 | 0.97 | 0.98 |
| Tomato paste | 27 | 430.7 (286.7) | 22 - 980 | 25 | 378.7 (345.5) | 10 - 1099 | 33 | 355.7 (296.9) | 17 - 1099 | 23 | 345.2 (340.3) | 9 - 1210 | 0.76 | 0.82 |
| Ready meals | | | | | | | | | | | | | | |
| Ambient ready meals | 48 | 344.9 (104.4) | 140 - 562 | 108 | 348.8 (111.4) | 115 - 811 | 112 | 408.0 (545.4) | 1 - 5900 | 114 | 328.3 (114.9) | 37 - 783 | 0.25 | 0.99 |
| Chilled ready meals | 29 | 231.6 (92.6) | 42 - 450 | 121 | 315.0 (115.7) | 84 - 679 | 116 | 306.4 (118.3) | 41 - 864 | 158 | 282.7 (119.9) | 41 - 828 | <0.01 | 0.20 |
| Frozen ready meals | 131 | 268.2 (55.5) | 129 - 435 | 207 | 268.9 (96.3) | 94 - 931 | 259 | 329.9 (170.4) | 60 - 1760 | 205 | 252.6 (75.5) | 84 - 595 | <0.001 | 0.70 |
| Dips | | | | | | | | | | | | | | |
| Chilled dips | 112 | 435.9 (189.9) | 15 - 1216 | 256 | 451.8 (235.2) | 39 - 2200 | 226 | 472.0 (210.3) | 104 - 2100 | 273 | 496.2 (249.0) | 5 - 1930 | 0.05 | 0.14 |
| Salsa | 9 | 560.0 (135.5) | 410 - 780 | 27 | 476.0 (124.5) | 5 - 645 | 26 | 457.0 (95.3) | 300 - 629 | 1 | 470.0 (-) | 470 - 470 | 0.15 | 0.91 |
| Savoury crackers | | | | | | | | | | | | | | |
| Plain dry crackers | 100 | 654.9 (283.4) | 2 - 1370 | 183 | 602.4 (342.6) | 0.18 - 2250 | 178 | 552.6 (239.7) | 2 - 1160 | 171 | 508.3 (218.6) | 1 - 1000 | <0.001 | 0.001 |
| Savoury crackers | 93 | 842.2 (293.2) | 220 - 1760 | 156 | 798.2 (289.9) | 173 - 1810 | 162 | 744.8 (289.8) | 173 - 2100 | 144 | 715.6 (275.6) | 281 - 2100 | <0.01 | 0.01 |
| Selected processed meats | | | | | | | | | | | | | | |
| Bacon | 46 | 1259.0 (243.1) | 680 - 1950 | 52 | 1161.0 (245.1) | 499-2170 | 56 | 1152.0 (323.0) | 597-2900 | 59 | 1047.0 (137.9) | 660-1490 | < 0.001 | <0.001 |
| Sausages | 81 | 651.2 (152.6) | 269 - 1120 | 89 | 635.6 (150.7) | 410 - 1100 | 58 | 710.5 (198.4) | 327 - 1600 | 81 | 691.1 (181.6) | 310 - 1170 | < 0.001 | 0.62 |
| Sliced meat | 86 | 1086.2 (358.3) | 120 - 2800 | 109 | 947.2 (316.0) | 72 - 1710 | 125 | 889.8 (282.6) | 0 - 1500 | 103 | 1008.7 (345.8) | 82 - 2800 | 0.03 | 0.44 |
| Asian-style sauces | | | | | | | | | | | | | | |
| Fish sauce | 2 | 6061.5 (3715.8) | 3434 - 8689 | 7 | 7003.9 (1880.2) | 4279 - 9115 | 6 | 6792.3 (1293.9) | 4279 - 7803 | 6 | 9636.7 (1243.8) | 7992 - 10882 | 0.03 | 0.14 |
| Oyster sauce | 5 | 3505.0 (534.3) | 2951 - 4377 | 11 | 3619.5 (636.3) | 2550 - 4918 | 11 | 3508.4 (787.3) | 1803 - 4443 | 14 | 3604.4 (806.5) | 1803 - 4770 | 0.98 | 1.00 |
| Soy sauce | 10 | 6097.0 (1189.2) | 3390 - 7619 | 28 | 5841.4 (1245.0) | 3390 - 8762 | 26 | 6003.8 (1171.0) | 3810 - 8762 | 31 | 6147.6 (1356.4) | 3808 - 8762 | 0.82 | 1.00 |

^a^ 2018 for Asian-style sauces only, 2017 for all other product categories

^b^ P-value the result of a one-way ANOVA

^c^ P-value the result of post-hoc Scheffe’s test following one-way ANOVA
